# Structure and crystallography of foliated and chalk shell microstructures of the oyster *Magallana*: the same materials grown under different conditions

**DOI:** 10.1038/s41598-018-25923-6

**Published:** 2018-05-14

**Authors:** Antonio G. Checa, Elizabeth M. Harper, Alicia González-Segura

**Affiliations:** 1 Departamento de Estratigrafía y Paleontología, Universidad de Granada, 18071 Granada, Spain; 2 Instituto Andaluz de Ciencias de la Tierra, CSIC-Universidad de Granada, 18100 Armilla, Spain; 3 Department of Earth Sciences, Cambridge University, Cambridge, CB2 3EQ UK; 4 Centro de Instrumentación Científica, Universidad de Granada, 18071 Granada, Spain

## Abstract

Oyster shells are mainly composed of layers of foliated microstructure and lenses of chalk, a highly porous, apparently poorly organized and mechanically weak material. We performed a structural and crystallographic study of both materials, paying attention to the transitions between them. The morphology and crystallography of the laths comprising both microstructures are similar. The main differences were, in general, crystallographic orientation and texture. Whereas the foliated microstructure has a moderate sheet texture, with a defined 001 maximum, the chalk has a much weaker sheet texture, with a defined 011 maximum. This is striking because of the much more disorganized aspect of the chalk. We hypothesize that part of the unanticipated order is inherited from the foliated microstructure by means of, possibly, ${\boldsymbol{\{}}{\bf{01}}\bar{{\bf{1}}}{\bf{8}}{\boldsymbol{\}}}$ twinning. Growth line distribution suggests that during chalk formation, the mantle separates from the previous shell several times faster than for the foliated material. A shortage of structural material causes the chalk to become highly porous and allows crystals to reorient at a high angle to the mantle surface, with which they continue to keep contact. In conclusion, both materials are structurally similar and the differences in orientation and aspect simply result from differences in growth conditions.

## Introduction

Oysters (Family Ostreidae) build thick shells out of composite calcium carbonates. The carbonate is predominantly in the form of calcite and the only aragonitic components are associated with the muscle attachment sites (myostraca) and fibres within the ligament^1^. The calcite is arranged into different microstructures. Although the bulk of both valves is made up of continuous layers of sheet-like foliae with a thin external layer of prisms (which is thicker on the right than left valve), shells are often characterized by discontinuous lense like intercalations of ‘chalk’^2,3^ (termed by Gray^4^). Whereas foliated calcite is a common (but not universal) development in pteriomorph bivalves and ubiquitous within the order Pectinida and the superfamily Ostreoidea^5^, chalk is unique amongst other molluscan microstructures and is found only in the Ostreidae. It is very distinctive, being composed of a loose array of crystals, made up of long laths (blades and leaflets) placed perpendicular to the growth surface, seemingly with a high degree of disorganization, and which is notably soft and porous (80% according to^6^).

Detailed descriptions of chalk morphology have been given in most studies concerning oyster shell structure (e.g.^4,7,8^) but little is known of its crystallography and mode of formation. This is unfortunate because an understanding of the formation will be important in considering its function, and at the moment these issues remain largely obscure. The peculiarity of chalk-filled lenses has spawned a number of different opinions as to its occurrence and function, with opinions split as to whether its formation is merely a symptom of either low or high calcium supply^6,9–12^, or whether it forms when the shell secreting mantle is in some way removed from the shell surface^13^. In the most extreme cases, chalk has been accounted for as a form of ‘remote mineralization’^14–16^ under the control of bacterial activity rather than of the oyster itself. Other studies have sought to investigate its physical and chemical properties compared to other calcitic microstructures, noting its low density, ‘softness’ and low resistance to chemical (both acid and protease) attack^17–19^. The present paper concentrates on characterizing the morphology and crystallography of oyster chalk with the aim of understanding its formation and relationship with the other microstructures and the secretory tissue. Resolution of this issue is a pre-requisite to understanding why oysters secrete this particular microstructure.

Notwithstanding some views^14–16^, most authors regard the chalk as normal shell layers deposited directly by the oyster itself, an observation underlined by the discovery that the isotopic signature of chalk in individuals was identical to that of the foliated calcite and lacking that would suggest either a photosymbiotic or chemosymbiotic bacterial origin^20^. Mouchi *et al*.^21^ have demonstrated that the biomolecular composition of the two microstructures is at least partially different, and Langlet *et al*.^22^ have identified the appearance of growth lines within the chalk but not their relationship with those in the rest of the shell. The blades mostly forming the chalk appear to resemble the laths of the foliated layer, but little is known about their crystallography, which is a crucial aspect in order to determine if the chalk is either a “modified” type of foliated microstructure or a completely different type of microstructure. The only previous crystallographic data^23^ show that the calcite *c-*axes of crystals in the two microstructures are differently orientated but not drastically so.

It is our purpose to conduct a more detailed investigation of oyster chalk to examine both its crystallography and growth line information and thereby to address the following questions in order to decipher how the chalk is manufactured and to give clues at to its function: are the laths of the chalk identical in nature to those of the foliated microstructure? If so, are they continuous between both materials? Are growth lines continuous across the foliated/chalk boundaries? If so, is there a change in their spacing?

## Results

### General organization of the foliated layers and the chalk

**Figure 1:**
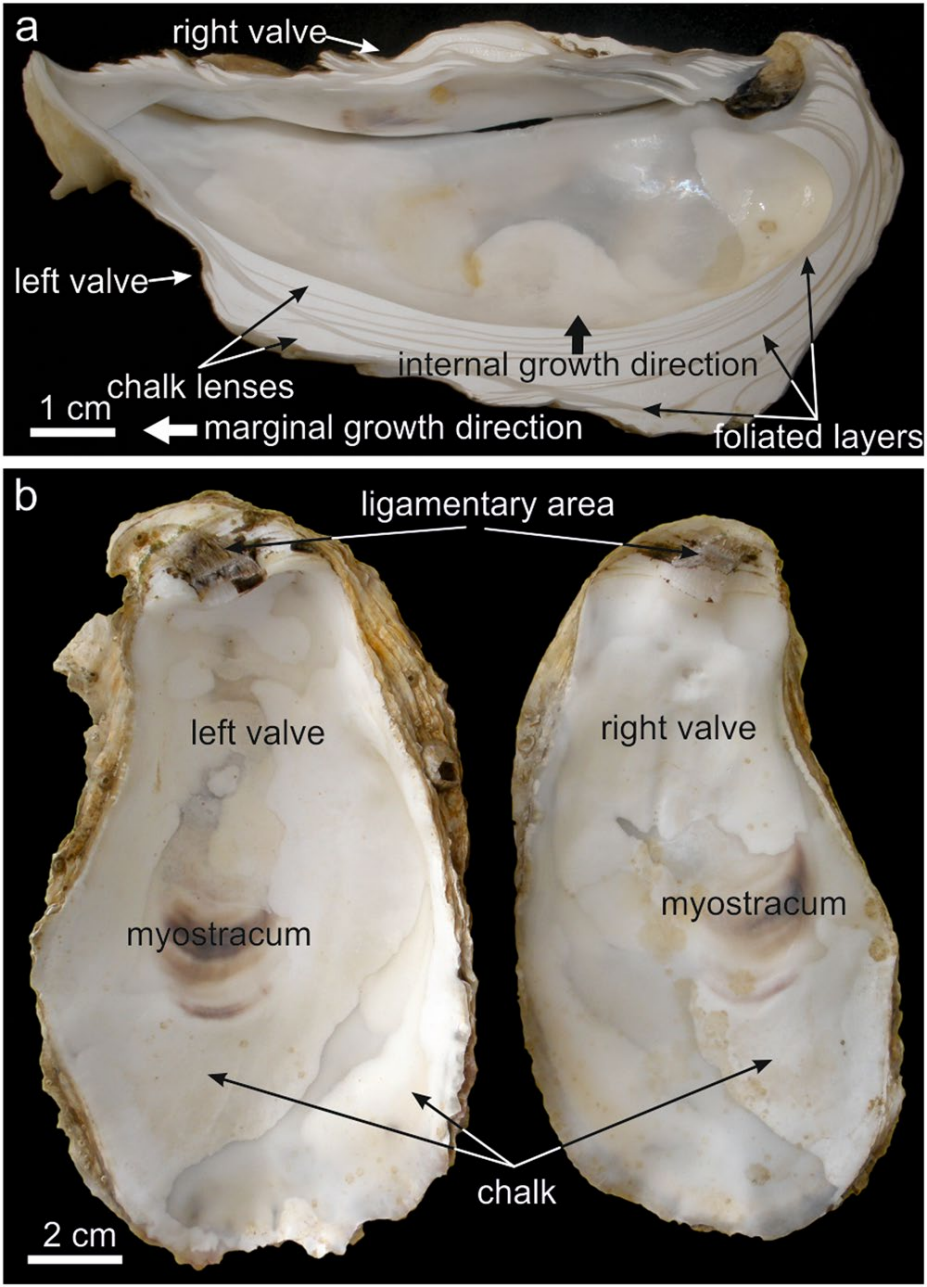
Distribution of chalk and foliated layers in the shells of *M*. *angulata*. (**a**) Dorsoventral section through a specimen; the chalk appears milky white, whereas the foliated microstructure is translucent grey. (**b**) Internal views of the valves of a specimen with a particularly wide and pseudosymmetric extension of chalk.

**Figure 2:**
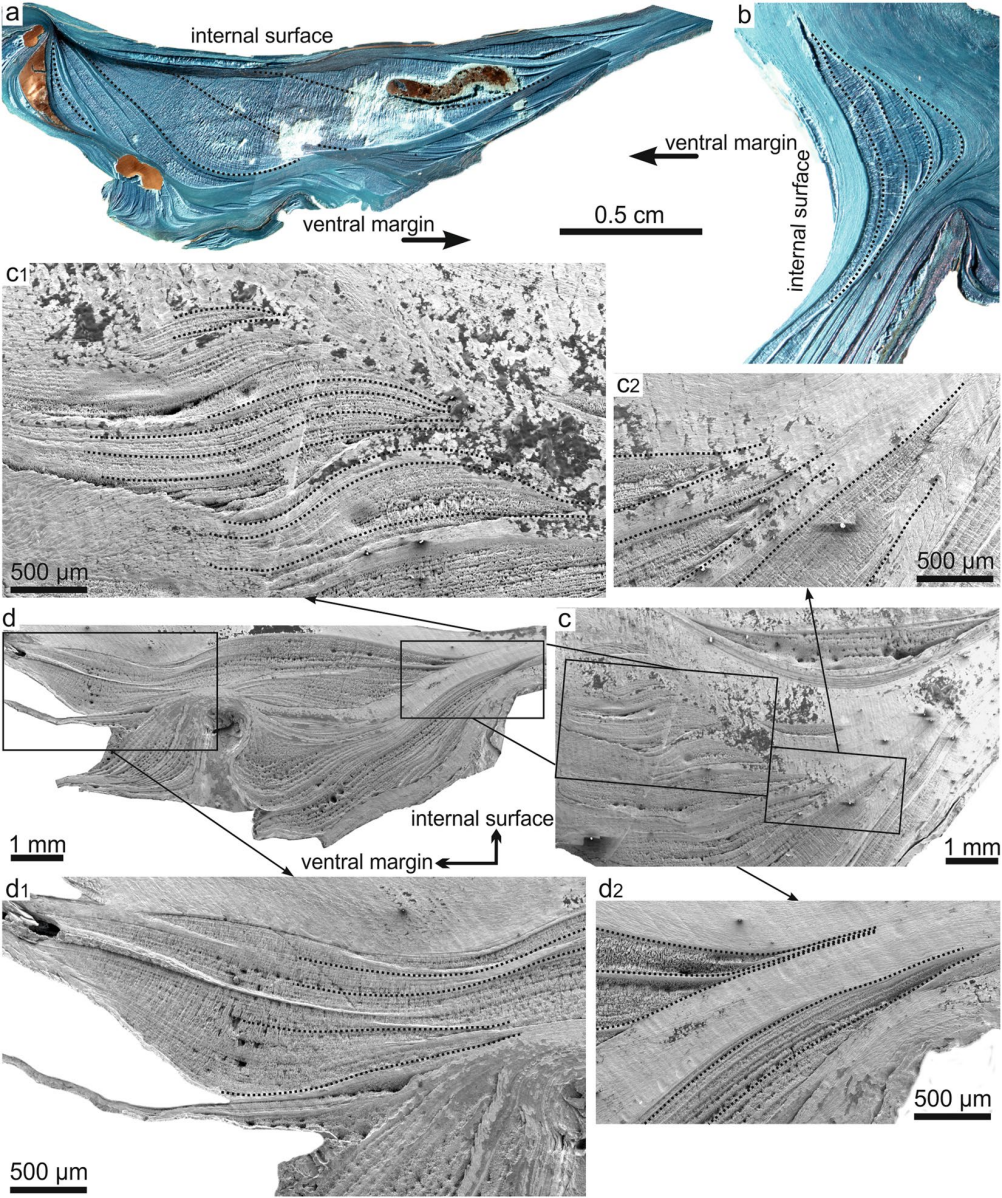
Polished and etched cross sections of left valves of *M*. *angulata*. (**a**,**b**) Composite optical micrographs of specimens treated with Mutvei’s solution. (**a**) Central part of a left valve. (**b**) Umbonal cavity of left valve. (**c**,**d**) SEM wide scope views of polished and etched specimens. (**c**) Central area. (**d**) Ventral area. c1, c2, d1 and d2 are close-up views of the areas framed in (**c**,**d**). Treatments reveal the outlines of chalk lenses, as well as the distribution of growth lines. Some growth lines have been outlined in order to show their continuity and the increase in spacing of the growth lines from the foliated material to the chalk.

**Figure 3:**
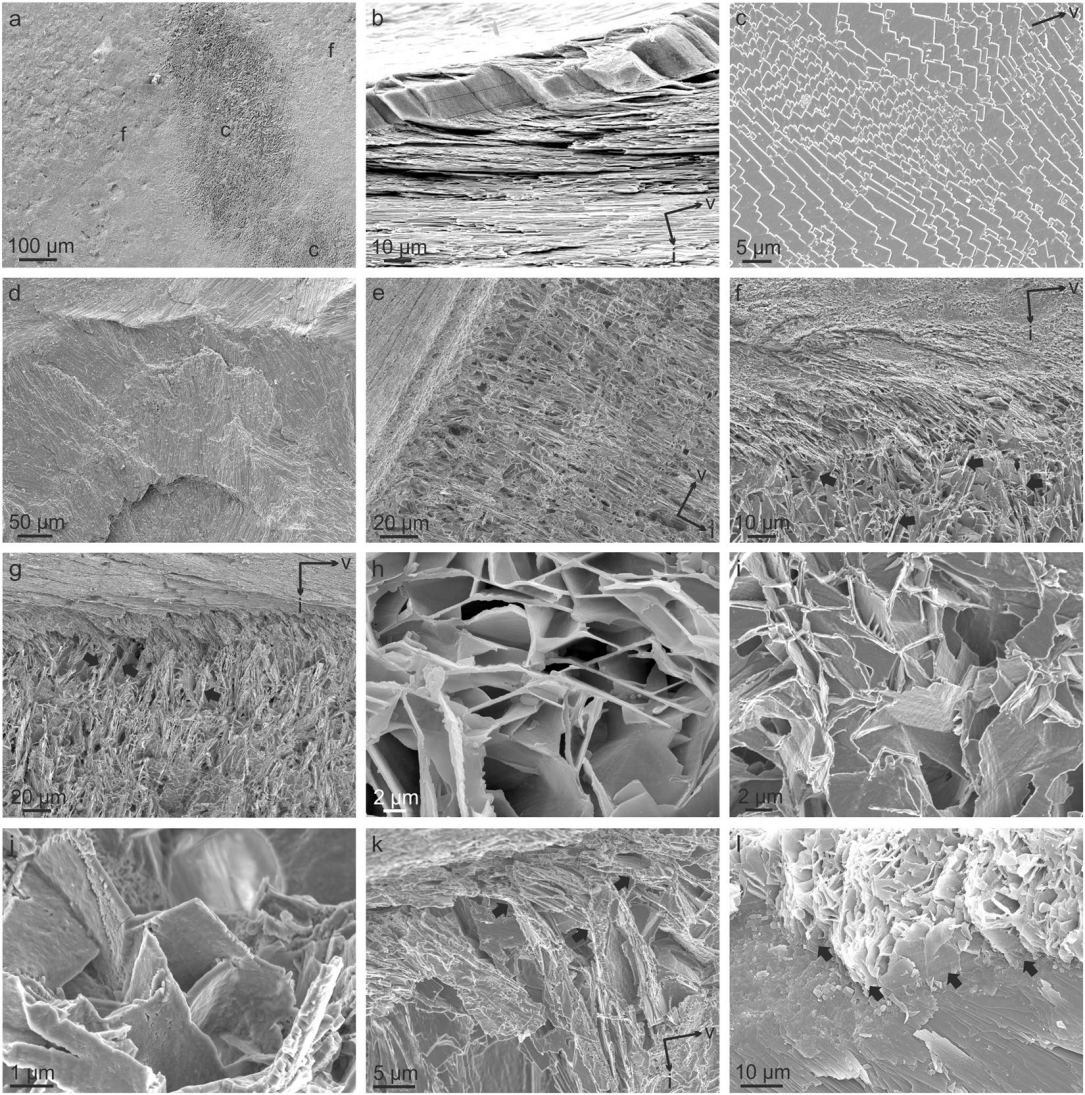
SEM views of microstructural details. (**a**) Chalk lens exposed on the inner surface of a left valve (f, foliated material; c, chalk). (**b**) Fractured right valve with outer prismatic and inner foliated layers. The inclination of laths with respect to the growth lines (some of them outlined with broken lines) is ~12°. (**c**) Foliated microstructure on the valve interior. Laths have varied orientations and their endings change from arrow pointed to straight. (**d**) Fracture subparallel to the valve surface showing bundles of laths in different orientations. (**e**) General view of the transition from the foliated microstructure (top left side) to the chalk. Blades are arranged roughly in perpendicular to growth lines. (**f**) Detail of the transition from the foliated microstructure to the chalk. At the transition, laths bend slightly towards the shell interior. Arrows point to some blades, apparently unrelated to laths of the foliated layer. (**g**) Detail of a transition similar to that in (**f**). In addition to bending, some laths deflect abruptly (arrows) at obtuse angles. (**h**) Detail of the chalk interior revealing consistent angular relationships between blades and leaflets. (**i**) Same as in (**h**) showing how the elements arrange polyhedrally. (**j**) Arrow point endings of some chalk elements. (**k**) Close up of the transition from the foliated microstructure to the chalk, showing abrupt changes in orientations of laths (arrows). (**l**) Surface view from the shell interior of the transition from the foliated microstructure to the chalk, showing sudden deflections of laths (arrows). All views are from *M*. *angulata*, except for (**b**) and (**c**) (*O*. *edulis*). Long arrows labelled ‘v’ and ‘i’ in (**b**), (**c**), (**e**), (**f**), (**g**) and (**k**) indicate the ventral and internal directions respectively.

The shells of oysters are made by an alternation of foliated and chalk layers, with the chalk deposits forming more or less elongated lenticular bodies (Fig. 1a). To the naked eye, the chalk appears milky white, while the foliated layers are translucent grey (Fig. 1). Some shells are almost free of chalk material, whereas in others, they may occupy most of the volume/area of the valves. Chalk deposits appear in both valves at almost any location (Fig. 1b), although they are most frequent in the umbonal cavity of the left valve (Figs 1a and 2b). Sometimes, there is some, though not strict correspondence between chalk lenses on both valves (Fig. 1b). Chalk lenses have most usually concavo convex outline (i.e. they are meniscus shaped), with the external boundary (i.e. that closer to the external shell surface) being more curved (Figs 1a and 2), although there are instances or biconvex sigmoidal lenses (Fig. 2a,c). Rare instances with less curved convex (even slightly concave or sigmoidal) external walls can be observed (Fig. 2c). Only seldomly and very locally, chalk deposits are exposed on the interior surfaces of the valves (Fig. 3a); otherwise, they are covered by, even if very thin, foliated layers. Chalk chambers are always isolated from the valve exterior by foliated material (Figs 1a and 2).

### Detailed microstructure

Both valves have a thin external layer of prismatic calcite (Fig. 3b) which is thicker on the right than the left valve^2,24^. Prisms are 5–25 µm wide and 20–50 µm high. As noted previously^25^, the prisms have themselves a foliated internal structure and grade into the foliated structure by loss of the organic sheaths around the prisms. The foliated microstructure, which has been described extensively by previous authors^2,26,27^, consists of planar arrangements (folia) of evenly-oriented longitudinal elements (laths), which meet the shell growth surface at a low angle of between 10–30° (Fig. 3b,c). Laths are typically 3–10 µm wide and some 200–250 nm thick and have arrow-point endings (Fig. 3b,c). The arrow point can be laterally displaced to a variable extent, and in some cases the edge becomes straight (Fig. 3c). Folia are evenly oriented only locally but the orientation may change both between different areas of the shell growth surface or in depth towards the shell interior (Fig. 3c,d).

**Figure 4:**
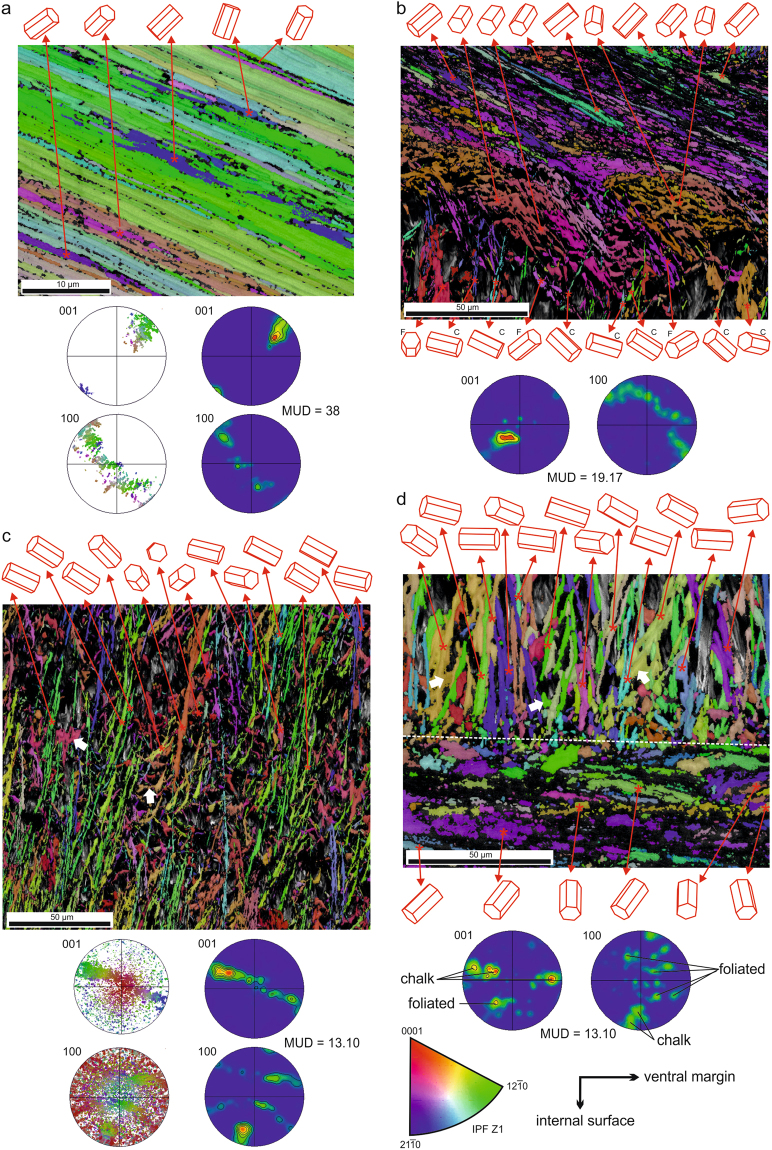
EBSD orientation maps, and corresponding pole figures of the shell microstructures of *M*. *angulata*. Orientations of the unit cells of particular laths are provided. (**a**) Foliated microstructure. (**b**) Transition from the foliated microstructure (top) to the chalk. Note angular features of laths in even colors. Laths with foliated-like crystallography are indicated with “F” and those with chalk-like crystallography are indicated with “C” in the lower row. (**c**) Chalk interior. Arrows point to leaflets diverging from blades, both in the same color. (**d**) Transition from the chalk to the foliated microstructure. Arrows indicate leaflets derived from blades. The broken line is the boundary between both materials. EBSD maps of (**a**) to (**d**) are superimposed onto the image quality maps. IPF, Inverse pole figure (orientation color key), valid for all maps.

As described elsewhere^3,8,28^, the chalk is made of thin (200–300 nm thick) and densely-spaced long blades, mainly perpendicular to the chamber surfaces, and leaflets (similar thickness), which stem at a high angle from the blades and extend between blades although sometimes not continuously (Fig. 3e–k). The elements are mainly straight, and only exceptionally curve locally. The morphological relationships are complex. In restricted areas, blades and leaflets may appear in parallel sets and keep consistent regular angular relationships between 135°–150° (Fig. 3h). In three dimensions, laths can be observed forming polyhedral features (Fig. 3i). Rare instances of leaflets with arrow-point endings have been observed (Fig. 3j). At the transition from the foliated layers to the chalk the laths of the former bend slightly towards the interior of the chalk (Fig. 3f) and later abruply deflect by very similar angles as above (130°–145°), sometimes more than once, to enter the chalk at a high angle to the growth surface (Fig. 3f,g,k,l). Soon within the chalk, new, apparently *de*-*novo*-formed laths begin to appear, which are characteristically subperpendicular to the surfaces of the chalk lenses (Figs 3f,g,k and 4b).

### Growth lines

In specimens treated with Mutvei’s Solution^29^ it is easy to see how the growth lines are continuous from the foliated material throughout the chalk (Fig. 2). The spacing between growth lines increases as it passes progressively from the foliated layer to the chalk; from 1.9 to 5.7 times (mean = 3.2 times, n = 15) their spacing in the foliated material (6–13.5 µm; mean = 10.1 µm; n = 15) (Fig. 2). This spacing becomes widest at the centre of the chalk chamber before becoming closer again towards the other end of the chamber. The pattern of separation of growth lines is progressive and depends very much on the shapes of the chamber boundaries. In external boundaries that are markedly convex towards the outer shell surface, progressively bigger extensions of the subsequently-formed (younger) growth lines become more spaced (e.g. Fig. 2d). The contrary is true for more convex internal boundaries (e.g. Fig. 2c). This effect diminishes with the flatness of the boundary.

### Crystallography

EBSD orientation maps of sections of the foliated layers transverse to the shell surface show that orientations (indicated by colors) change across the shell thickness (Fig. 4a,b). Superimposed evenly colored folia appear isolated or in sets of up to 7–8. Rarely, a few folia with a different orientation appear sandwiched within a bigger pack (Fig. 4a). Misorientations within individual laths are of the order of 2–10° (Supplementary Fig. S1a). Raw pole figures show an appreciable degree of spread (Fig. 4a). Density pole figures show well individualized maxima, although their MUD values^30^ (see Methods) are small (38 in Fig. 4a; >30 in other maps made on foliated layers, not shown). There is a single wide 001 pole maximum placed at about 70°–75° (±20°) to the main elongation of laths (Fig. 4a,b). The mean measurement for individual laths was 71.6° (standard dev = 9.99; n = 10). The 100 pole figure reveals three loosely defined maxima (Fig. 4a,b). This implies that the superposed folia have their *c*- and *a*-axes roughly, though not strictly, co-oriented.

**Figure 5:**
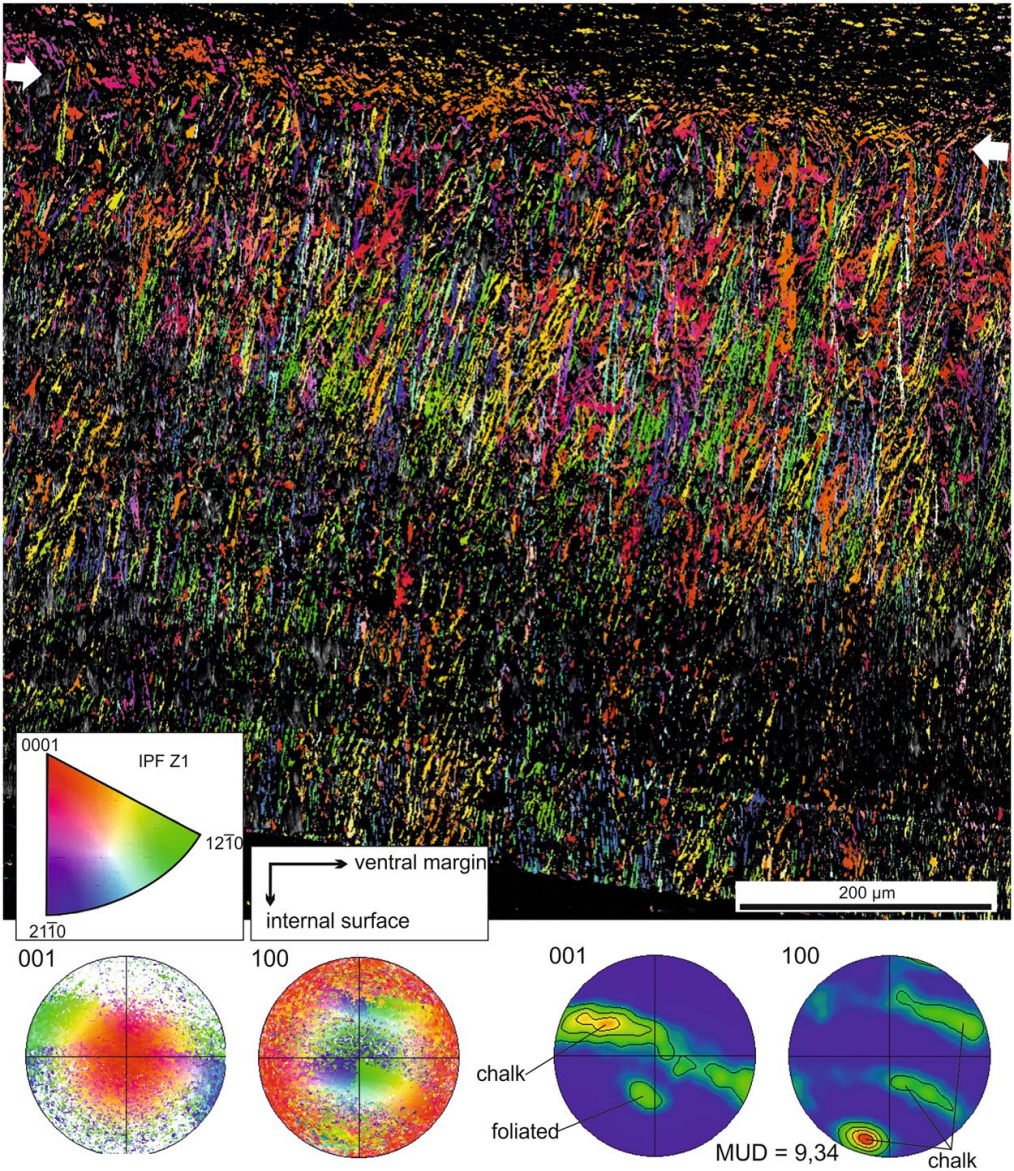
Orientation map covering the complete thickness of a chalk lens. The arrows indicate the boundary with the foliated layer (top). The pole figure maxima corresponding to either material are indicated. The orientation map is superimposed onto the image quality map. IPF, Inverse pole figure (orientation color key).

EBSD maps of transversely sectioned chalk interiors show laths in in varied orientations, i.e. in very different colors in the EBSD maps. Interestingly, there are many instances which reveal that blades retain their crystallographic orientation during bending (Fig. 4c,d). Likewise, blades and the leaflets diverging from them are in exactly the same colors (Fig. 4c,d, arrows), i.e. they are single crystals. Misorientation values within single blades are similar to those of the laths making up the foliated layer (Supplementary Fig. S1b). Raw pole figures are difficult to interpret due to the high degree of scattering of the poles (Figs 4c and 5). The 001 pole figure density plots show that the poles arrange in a sort of broad equatorial band inclined at roughly 75–80° with respect to the main orientation of the blades (Figs 4c,d and 5). The distribution of poles within this band is not even, and there is an elongated maximum placed approximately in the direction of the ventral margin (Figs 4c and 5). A similar distribution of 001 poles was obtained by X-ray diffractometry on the chalk of *Magallana gigas*^31^. Measurements of the inclination of the *c*-axis on individual blades provided a mean of 76.7° (standard dev = 5.1; n = 13). The distribution of the 100 poles is also diffuse, but not random. The pole density plots reveal a pattern with one of the maxima being relatively discrete and at a low angle (10°–20°) to the mean elongations of the blades (Figs 4c and 5). The other two 100 maxima take the form of arches, centred around the discrete maximum. MUD values are always much smaller (about one third) than those obtained from the foliated layers (13.1 in Figs 4c, 9.3 in Fig. 5).

At the initiation of the chalk from the foliated microstructure (i.e., foliated external, chalk internal), it is easy to see how the laths of the foliated layer and their angular deflections (observed with SEM; see above) have exactly the same crystallographic orientations (Figs 4b and 5). At the same time, other blades, with the crystallography above described for the chalk and seemingly unrelated to laths of the foliated layer, begin to emerge at very high angles to the foliated-chalk boundary. The change from one crystallography to another is seemingly sharp, with the 001 maxima belonging to both materials being totally separated (Fig. 5). By selecting only the elements with their 001 poles in an orientation similar to those of the laths of the foliated layer, we can observe some similar orientations throughout all the chalk thickness. However, these loci are very sparsely and randomly distributed (Supplementary Fig. S2) such that the coincidences may be due to the broad orientational density distribution in the chalk.

When secretion of chalk ceases and growth of the foliated microstructure resumes, there is a sharp boundary, without any clear transition (Fig. 4d). By selecting only the elements with a blade-like crystallography (equatorially distributed 001 poles), we observe that some blades go into the foliated layer, but for a short distance (Supplementary Fig. S3). As described above, the equatorially-distributed 001 poles of the chalk are totally separated from the discrete 001 maximum of the foliated microstructure at the chalk-to-foliated transition (Fig. 4d).

## Discussion

Our data allow us to firmly reject hypotheses that suggest that chalk is a form of remote mineralization caused by bacteria in empty shell chambers^14–16^ or that it relates to dissolution of shell caused in unfavourable environmental conditions^9,11^. Rather, we confirm that chalk is an integral part of the shell, deposited by the oyster itself as part of its normal repertoire of shell secretion, as suggested by previous authors (e.g.^12,32^).

Our results allow us to compare the crystallographic relationship between the chalk and foliated microstructures and also to provide a model to understand how the lenses of chalk are formed. Lastly, such an understanding provides a framework to start to understand the function of chalk within the shell.

### Crystallographic relationship between foliated and chalk microstructures

Our results show distinct similarities between the foliated and the chalk microstructures. Both are made of laths having the same thickness and aspect. Even some arrow-point endings have been observed within the chalk (Fig. 3j). The difference is that the laths of the foliated microstructure are closely packed and dip at a relatively low angle towards the growth surface, whereas chalk blades are sub-perpendicular to the growth surface and widely spaced within the chalk. The leaflets extend between the blades and diverge from them at varied angles.

Crystallographic data (pole figures and orientation maps) provide a wealth of information about the nature of both materials. EBSD 001 pole figures obtained on the foliated calcite indicate that there is some preferential, though not strict, orientation for the *c*-axis (Fig. 4a). The presence of maxima on the 100 pole density plots show that this is also the case for the *a*-axes. According to the pole figure distribution, the foliated calcite has a moderate to weak sheet texture, i.e. all axes are cooriented. This is true for the mapped thicknesses, of the order of several tens of µm. MUD values are relatively low, which matches with the high degree of spread observed in the raw pole figures (Fig. 4a).

Earlier authors^2,33,34^, using different techniques on internal shell surfaces (and not on sections, as in this study) of foliated microstructures, concluded that the main surfaces of laths are $\{10\bar{1}8\}$ rhombohedral faces of calcite, which are exclusive to the biogenic foliated calcite of bivalves of the orders Ostreida and Pectinida (Pteriomorphia). Alternatively, it was found that the same surfaces were of the $\{01\bar{1}8\}$ type in the families Pectinidae and Propeamussiidae of the Pectinida^33^. Our data do not precisely match these conclusions. First, the mean values encountered (70°–75°) between the elongations of laths and the *c*-axes do not correspond with those of either the $\{10\bar{1}8\}$ or $\{01\bar{1}8\}$ faces (63.24°), but rather argue for higher values of the *l* Miller index, between 10 (i.e. $\{10\bar{1}\,\cdot \,10\}$ or $\{01\bar{1}\,\cdot \,10\}$, whose angle to the *c*-axis is 68.45°) and 15 ($\{10\bar{1}\,\cdot \,15\}$ or $\{01\bar{1}\,\cdot \,15\}$, angle to *c*-axis, 75.25°). Second, the wide range of angles for a consistent orientation of laths (see orientation map and raw pole figures in Fig. 4a) also indicates that attribution to a particular rhombohedron is not possible, with the actual range of values being much wider than that suggested previously.

According to the Bravais rule, the importance of faces in a crystal decreases with increasing value of the Miller indices because of the decrease in the density of atoms and stabilizing bonds parallel to the crystallographical planes. Such rhombohedral faces are very high-energy^35^ and never develop in inorganic calcite. Likewise, they are characterized by very low densities of calcium atoms and carbonate groups (Supplementary Fig. S4). Additionally, an irregular surface (as is the case of the main surfaces of laths; Supplementary Fig. S5) has a higher energy than a flat surface because it has more disturbed bonds. We hypothesize that those irregular surfaces appear in biogenic calcite because their very low saturation of bonds favours weak bonding interactions with organic molecules. Their adhesion decreases the surface energy and promotes stabilization of these surfaces. Accordingly, they cannot be considered as true crystal faces, but as the surfaces left behind by the $\{10\bar{1}4\}$ surfaces forming the arrow-pointed growth tip of laths, whose further growth is inhibited by the adhesion of organic molecules; i.e., they have to be better understood as inhibition surfaces. This fits in with their wide range of orientations and with their roughness, compared to the actual $\{10\bar{1}4\}$ faces (Supplementary Fig. S5).

The attribution to such flat rhombohedra is consistent with the observed recurrent orientation relationships between blades, as well as blades with leaflets, at wide angles (Fig. 3h). Since EBSD proves that the crystallographic orientation does not change during the deflection or divergence (Fig. 4c,d), the elements in this angular relationship can be interpreted (in a way similar to the foliated material, above) as belonging to different surfaces of the same skeletal rhombohedron. Accordingly, the reported differences in the kind of biomacromolecules between both the foliated and chalk materials^21^ are not reflected in the crystallographic structure.The angular deflections of laths of the foliated microstructure at the transition to the chalk with invariant orientations (Fig. 4b) are clear cases of laths running along different surfaces of the same rhombohedron, which would in this way be of the skeletal (hollow) type.

Compared to the foliated material, the chalk appears more disordered (MUD values are about one third those of the foliated calcite), but still shows some degree of order (Figs 4c,d and 5). The 001 pole density plots (i.e. the *c*-axes) have a ring-like distribution, with some degree of preferential concentration in the area pointing towards the ventral margin. The *a*-axes also show preferential orientations, with one 100 maximum (the one closest to the lower end pole of the pole figures) being only very slightly elongated, i.e. close to the common or fibre axis, around which the two other 100 maxima rotate. This pattern can be categorised as a fibre texture, but it differs from that recognized in other biogenic calcites (e.g. the columnar prismatic calcite of bivalves^23,36^, and the fibrous calcite of terebratulid brachiopods^37,38^) where the *c*-axis is the fibre axis. If we take into account the weak elongated maxima observed in the ring-like distribution of the two other 100 poles as well as in the 001 poles, the texture of the chalk could even be categorised as a very weak sheet texture, which is surprising in view of the highly disoriented appearance of the material under the SEM.

Within the chalk, the range of orientations of the main elongation of blades with respect to the *c*-axes is very wide (see raw pole figures in Figs 4c and 5), but not significantly dissimilar to that of the foliated calcite. Accordingly, the laths of both the chalk and the foliated microstructure most likely have the same crystallography. Similarly to the foliated microstructure, the blade and leaflet main surfaces correspond to skeletal rhombohedral surfaces, averaged around $\{10\bar{1}\,\cdot \,15\}$ or $\{01\bar{1}\,\cdot \,15\}$.

**Figure 6:**
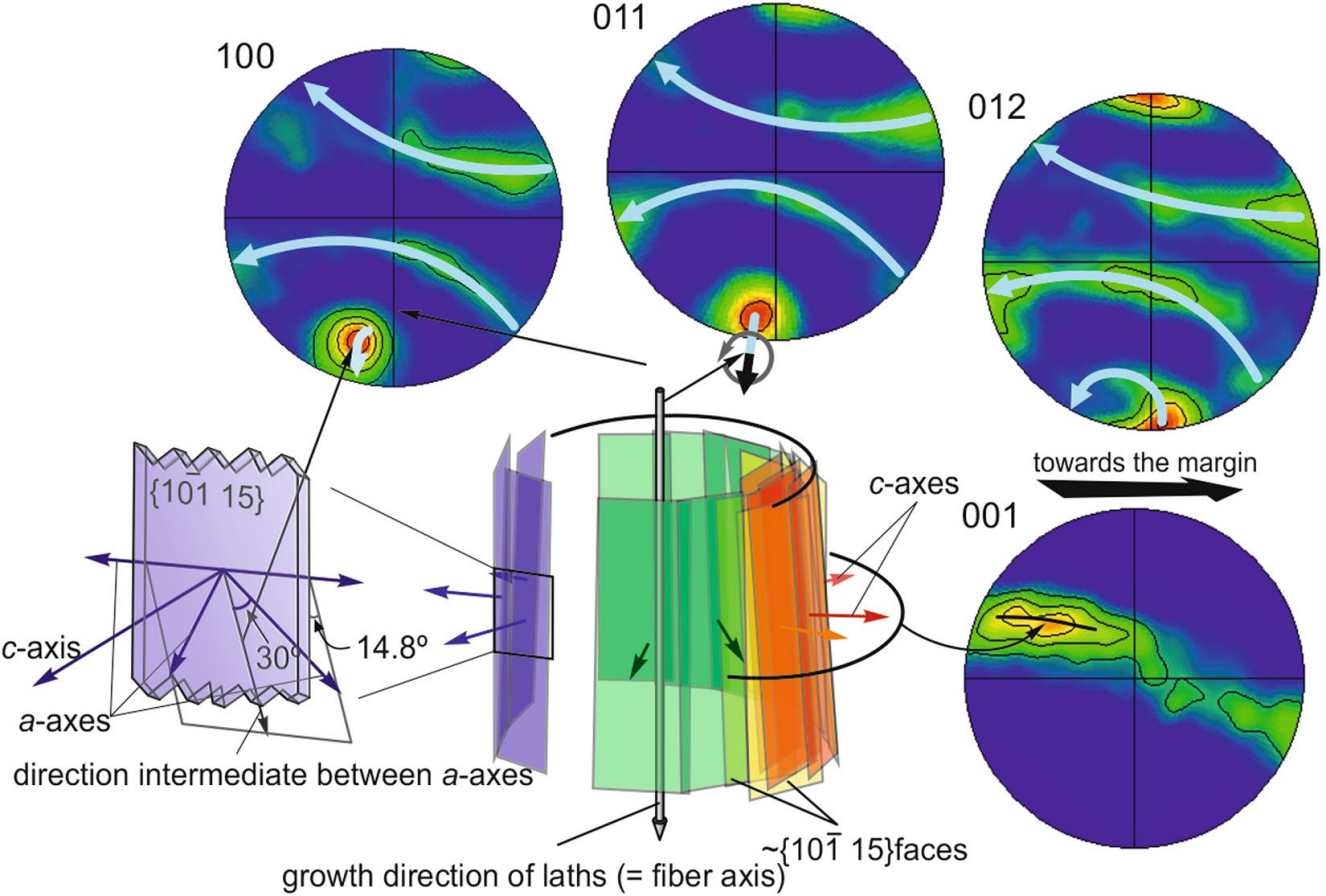
Crystallographic structure of the chalk, according to the distribution of pole maxima. Blades have a cylindrical distribution with the fibre axis being the growth axis of blades. These are in turn almost coincident with the 011 poles. Blades are more densely distributed in the direction of the margin. 100 and 001 pole figures adapted from Fig. 5.

In calcite, the 108 poles are almost perpendicular to the 012 poles (namely 89.59°) (Supplementary Fig. S6), which implies that the directions perpendicular to $\{01\bar{1}2\}$ are subparallel to $\{10\bar{1}8\}$. Accordingly, the 012 poles represent the orientations of directions virtually contained within $\{10\bar{1}8\}$ faces. The same applies to the 10 15 and 011 poles (separated by 90.58°; Supplementary Fig. S6). If we compare the 100, 011 and 102 pole figures in Fig. 6, it is clear that the lower 011 maximum is the one with the least spread, i.e., it is the closest to the fibre axis of the chalk. Since the directions corresponding to the 011 poles are virtually contained within $\{10\bar{1}\,\cdot \,15\}$ faces, which correspond to the mean orientations of the main surfaces of blades, we hypothesize that the fibre axis is approximately coincident with the growth axis of the blades. This is consistent with the aspect of the chalk in sections, where the blades are subperpendicular to the growth lines (Figs 3e and 5). Given the fibre-like texture observed, the laths should have a cylindrical distribution. According to the 001 and 012 pole figures (Fig. 6), laths are more densely distributed in the zone looking in the marginal direction. This structural model is depicted in Fig. 6.

Although the laths initially cross the foliated-chalk boundary while retaining their crystallographic orientations, both SEM (Fig. 3f) and EBSD (Fig. 4b) images show that very soon within the chalk, laths with ‘chalk’ type orientation (i.e. blades) begin to appear. Most possibly, they nucleate *de novo* within the empty spaces typical of the chalk. Their fast proliferation explains the sudden change in the crystallographic pattern revealed by pole figures.

Despite the weak texture of the chalk, it is striking that both the 001 and 100 poles cluster around some maxima, which reveals some degree of order. How this order arises cannot be explained if blades do not keep any crystallographic relationship with the laths of the previously formed foliated layer onto which they nucleate, i.e. unless orientations are somehow ‘inherited’ from the preceding foliated layer. A likely possibility is twinning, which would allow blades to consistently change their orientations. We have checked this possibility by obtaining the pole figures corresponding to three of the four twin planes described in inorganic calcite: $\{10\bar{1}4\}$, $\{01\bar{1}2\}$, $\{01\bar{1}8\}$^39^, as well as the $\{10\bar{1}8\}$ twin exclusive of biogenic calcite^40^ (Supplementary Fig. S7a). The remaining twin law, {0001} has not been checked because it would not allow the laths to change the orientation of their *c*-axes^39^. Then, we selected only those crystals (both from the foliated layer and the chalk) with their poles at or close to the most conspicuous maxima of the pole figures (Supplementary Fig. S7a). In this way, we can check if the laths of both the foliated microstructure and the chalk putatively share a particular plane. Cropping of 104, 012, and 108 maxima always selects a high number of crystals in different colors, which argues for the putative existence of such twins, but only within the chalk (Supplementary Fig. S7b). Only when the maximum in the 018 pole figure is cropped, crystals from both materials become selected, which suggests $\{01\bar{1}8\}$ twinning relationships (Supplementary Fig. S7c). An ideal $\{01\bar{1}8\}$ twin brings about a rotation of the *c*-axes of 126.48° around one of the *a*-axes. The angular distance between the 001 maxima of the foliated material and the chalk in our largest map (Fig. 5) is 65°–75°, which is not far from the angle supplementary to the above. In summary, we hypothesize that the crystallographic orientation of the chalk is, to some extent, inherited from the preceding foliated layers by $\{01\bar{1}8\}$ twinning.

### Model for chalk formation

Since the units forming both the foliated layers and the chalk are basically the same, the differences observed in the orientation and density of the elements between both materials can to be attributed to the different spacings of the growth lines. Langlet *et al*.^22^ showed the existence of growth lines within the chalk and mentioned their wider spacing, but they did not provide a clear image of the processes taking place at the transition with the foliated layers. We have observed that growth lines are continuous between the foliae and the chalk and how they become progressively wider apart at the transition from the foliated to the chalk microstructure (Fig. 2). Thus, it is clear that both materials are made simultaneously by different parts of the shell secreting mantle, but the mantle must separate from the formerly laid down shell several times faster during chalk formation than that of the foliated microstructure. Accordingly, the differences can be traced back to differences in the conditions created by the mantle of the animal during the construction of both materials.

**Figure 7:**
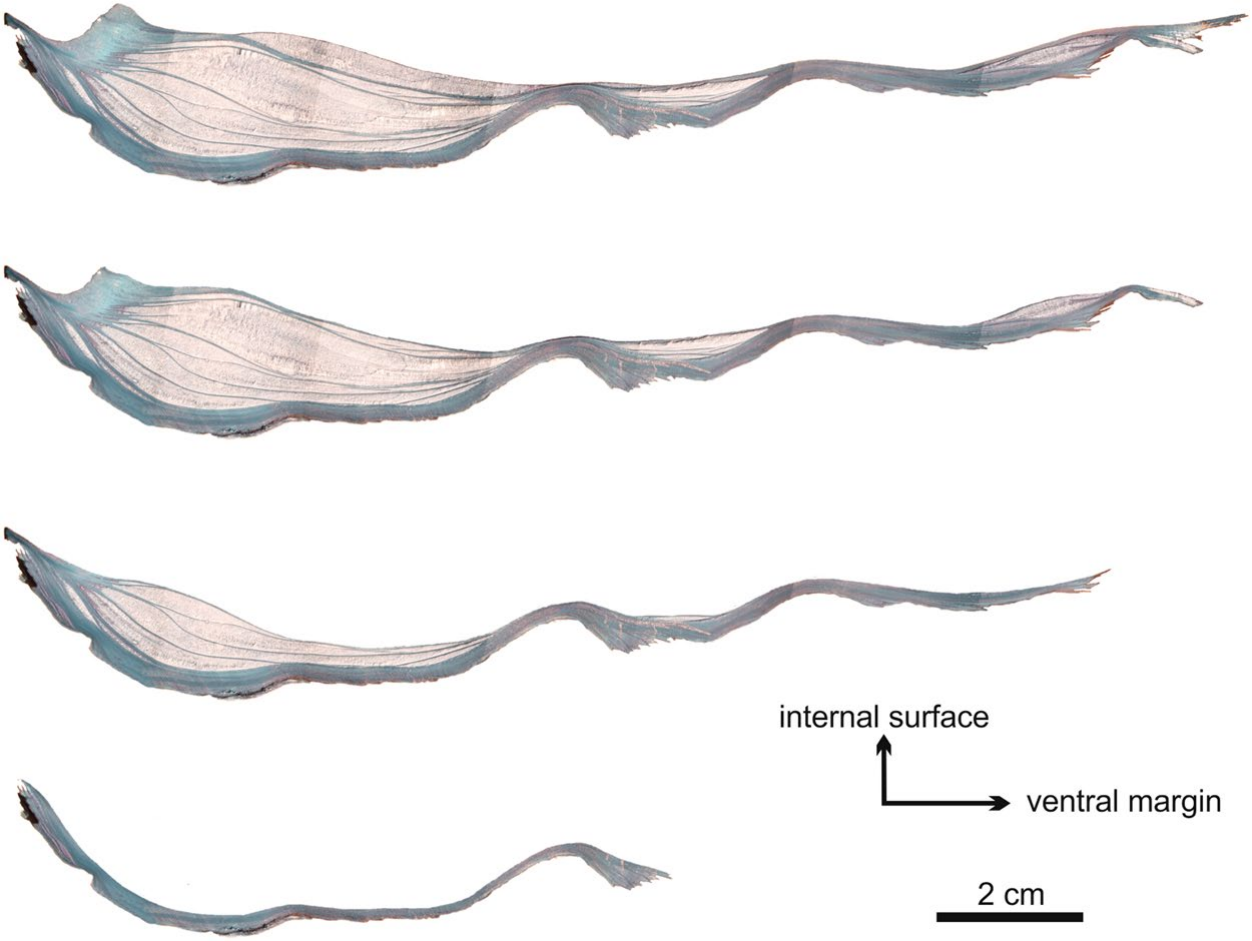
Successive growth stages of the left (lower) valve of one individual of *M*. *angulata*. The reconstruction has been made by back-stripping growth episodes along prominent growth lines.

During the secretion of the foliated microstructure the mantle is separated from the growth fronts of crystals by the extrapallial space (shown to be extremely thin in other bivalves^41^) and moves away slowly enough to provide construction material for all tightly packed laths. With the increase in the rate of separation of the mantle during chalk production, this trade-off is lost and the mantle is not able to produce the necessary amount of calcite precursors (ions or calcium carbonate nanoaggregates) to fill the excess of volume. Due to the availability of empty volume, the crystals are able to change their orientations (already at the contact foliated-chalk), first bending and later becoming subperpendicular to the growth lines. This orientation provides the shortest path in order for the growth fronts (their most internal surfaces) of blades to keep contact with the shell-secreting outer mantle surface. This is similar to what happens in inorganic aggregates, in which the crystals tend to grow perpendicular or at a high angle to the substrate, with the difference that their growth surfaces are free and not limited by the mantle, as in the chalk. Evidence that blades retain contact with the mantle is provided by the presence of growth lines within the chalk. During blade growth, leaflets stem from them with the same crystalline orientation, until they are intercepted by other blades or stop growing some way in between. It is doubtful that they always grow in contact with the mantle, this being particularly true for subhorizontal leaflets, i.e. some could be partly remotely secreted. Figure 7 shows the way in which the spacing of the growth lines varies, and how they are widest over chalk-filled areas, by showing the successive growth phases of a single valve.

Chalk is exceptional among molluscan microstructures in its high final volume of intergrain porosity. Only growing gastropod nacre shows empty spaces between the growing nacre towers, although the material is wholly compartmentalized by horizontal organic membranes and the fully-grown product is well ordered and tightly packed. As a consequence of its porosity, the degree of ordering of the chalk (largely inherited from the foliated layers) is the lowest found hitherto (compared to other MUD values provided in^42^). This is a good example of how the normal balance between mineral secretion capacity of the mantle and shell thickening rate (i.e. volume restriction) contributes to the high degree of order in molluscan microstructures.

The vesicular microstructure developed by some members of the Gryphaeidae (grouped with the Ostreidae under the Ostreoidea) is also a lightweight and highly porous structure. Nevertheless, it consists of columnar hollow cavities with thick walls made of small granules of calcite (E. M. Harper and A. G. Checa, unpublished observations). Therefore, it is totally unrelated to the chalk.

### Implication for understanding any functional value of chalk

Although our study deals only with fabricational aspects of chalk formation it does allow us to comment on some of the myriad of functional explanations that have been proposed for the chalk. The idea that chalk is formed when the mantle is separated at a fast rate from the growing surface lends support to the notion that chalk may form as a way of smoothing out the interior of the shell from irregularities imposed by an irregular substrate^6,12,13^. This appears to be illustrated, for example by the individual shown in Fig. 7. In this way it may be hypothesized that oysters which encrust more regular surfaces will be less inclined to chalk formation. If this is so then other proposed advantages of chalk microstructure (e.g. low density^43^) must be seen as secondary or exaptive^44^.

## Methods

### Material

Several tens of dead shells of the Portuguese oyster *Magallana angulata* (formerly known as *Crassostrea angulata*, until recently placed in the new genus *Magallana*^45^) were sampled from shell middens at the land-facing margin of the Formosa inlet immediately adjacent to the locality of Cacela Velha (S. Portugal) and from a similar setting at the neighbouring location of Ilha de Tavira. Only shells with their valves still articulated and in a good state of preservation were selected for study. Dorsoventral diameters ranged between 60 and 156 mm. Fresh empty left valves, likely belonging to the Pacific oyster *Magallana gigas* (possiby conspecific to *M*. *angulata*^46^), were provided at the Loch Fyne Restaurant (Cambridge, UK). Additional observations were made on specimens of *Ostrea edulis*, acquired alive in markets in Granada (Spain).

### Etching and staining protocols

We selected ten particularly well preserved specimens with articulated valves from the Formosa inlet. The valves were stuck together with cyanocrylate glue, and sectioned perpendicular to the valve surfaces and close to the dorsoventral axis. After resin-embedding, we cut a slice about 1 cm thick, one of whose main surfaces run as precisely as possible along the dorsoventral axis. The exposed surface was polished with carborundum up to a grit size of 1000. The slice was subjected to treatment with Mutvei’s Solution in order to improve visualization of the growth lines. The procedure is described in detail in^29^, but briefly the solution contains identical volumes of 1% acetic acid and 25% glutaraldehyde, plus 1 g alcian blue powder per 100 ml of solution. Samples were immersed for 25 min at 40 °C, under continuous stirring and ensuring that the polished surfaces were always in contact with the solution. The stained samples were then photographed with a Nikon SMZ 1000 binocular microscope.

### Scanning electron microscopy

Ultrasonicated fractures and internal surfaces, as well as polished sections were prepared for scanning electron microscopy (SEM) observation. Two Mutvei’s-treated specimens were cut into pieces with a Multi-Dremel cutting disc, ultrasonicated and air dried. All samples were carbon-coated (Emitech K975X carbon evaporator) and observed in a field emission SEM (FESEM) in either the Center for Scientific Instrumentation (CIC) of the University of Granada (Zeiss Auriga or FEI QemScan 650 F), Spain, or the University of Cambridge (Fei Qemscan 650 F), UK. Whilst characterising the individual microstructure types, particular attention was given to the transition between them.

### Electron backscatter diffraction

Two samples of the left valves of *M*. *gigas* were prepared for electron backscatter diffraction (EBSD). Fractures approximately perpendicular to the shell surfaces and containing both foliated microstructure and chalk were polished on horizontal diamond-impregnated discs (Struers Planopol 2 polishing machine) with grit sizes 3, 1 and 0.75 µm and followed by a final polishing with colloidal silica. To reduce charging, samples were coated with a thickness of 2 nm of carbon in a Baltec MED 020 electron beam evaporator (CIC, Univ. Granada). Analyses were carried out in the Zeiss Auriga Cross Beam work-station (operated at 20 kV) equipped with an Oxford Instruments Nordlysnano EBSD detector of the same center. Given the thinness of laths, the step size was reduced to 0.1 µm, except for the large map in Fig. 5 (step size, 0.25 µm). Data were post-processed with the analysis software HKL CHANNEL5 (Oxford Instruments) and is presented in the form of orientation color maps and pole figures. No grain dilation or averaging was applied. Multiples of uniform density (MUD) values have been calculated for sets of pole density figures. These values represent the strength of the clustering of poles, relative to that of a random distribution, or, in other terms, they are proportional to the degree of co-orientation of crystals^30^.

## Electronic supplementary material

Supplementary figures S1 to S7
