# Supplementary material for: Structure and crystallography of foliated and chalk shell microstructures of the oyster Magallana: the same materials grown under different conditions
Source: Sci Rep. 2018 May 14;8:7507. doi: 10.1038/s41598-018-25923-6 (PMC5951953; doi:10.1038/s41598-018-25923-6)
Supplement: Supplementary file 1 — Supplementary figures S1 to S7 [file 41598_2018_25923_MOESM1_ESM.pdf]

Structure and crystallography of foliated and chalk shell microstructures of the oyster *Magallana*: the same materials grown under different conditions

Antonio G. Checa[1,2], Elizabeth M. Harper[3] and Alicia González-Segura[4]

[1]Departamento de Estratigrafía y Paleontología, Universidad de Granada, 18071 Granada, Spain, [2]Instituto Andaluz de Ciencias de la Tierra, CSIC-Universidad de Granada, 18100 Armilla, Spain, [4]Department of Earth Sciences, Cambridge University, Cambridge CB2 3EQ, UK, [4]Centro de Instrumentación Científica, Universidad de Granada, 18071 Granada, Spain.

Supplementary figures S1 to S7

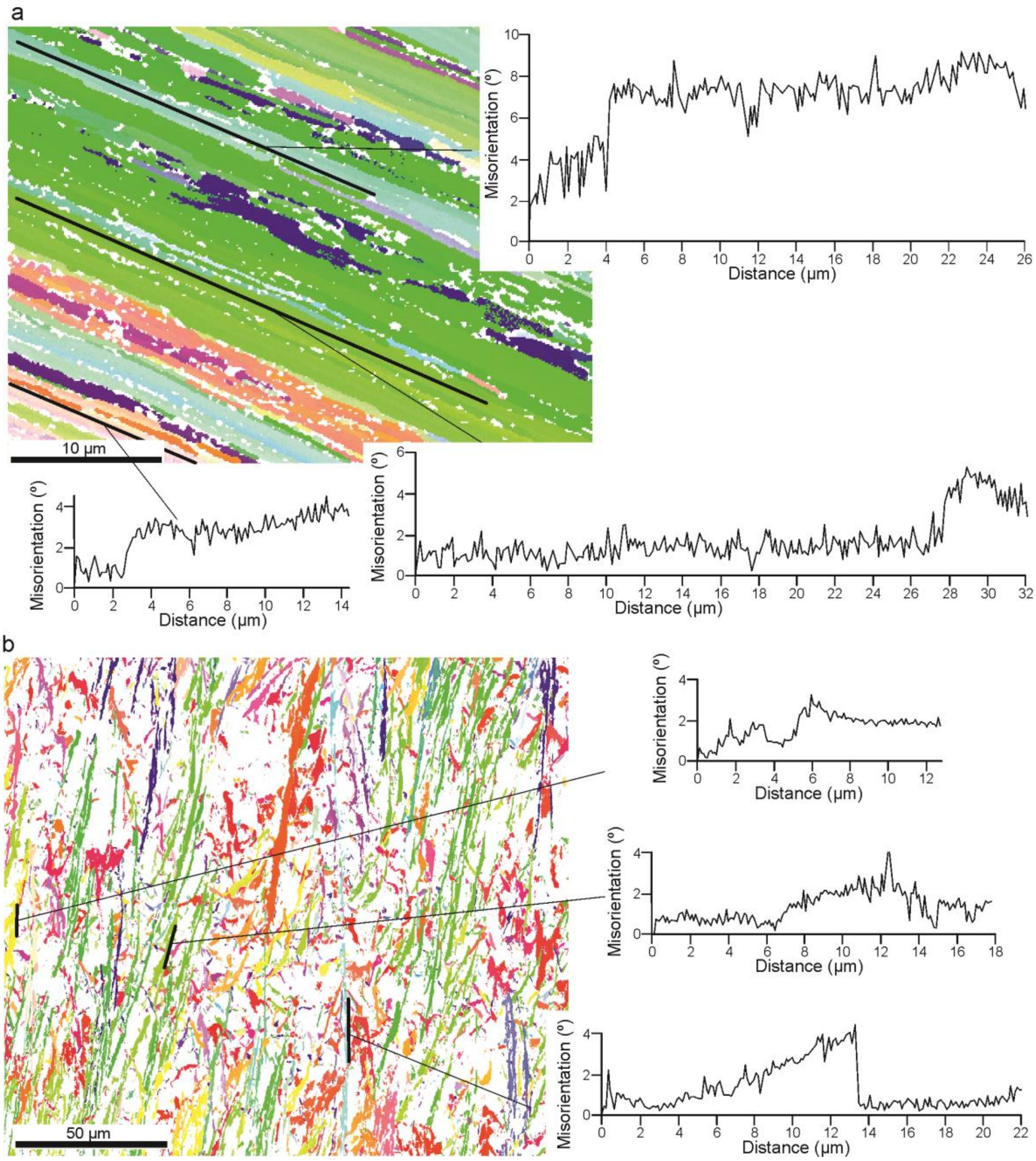

Supplementary Fig. S1. Misorientation profiles across individual laths. (**a**) Foliated layer. Same map as in Fig. 4a. (**b**) Chalk. Same map as in Fig. 4c. Color keys as in Figs 4 and 5.

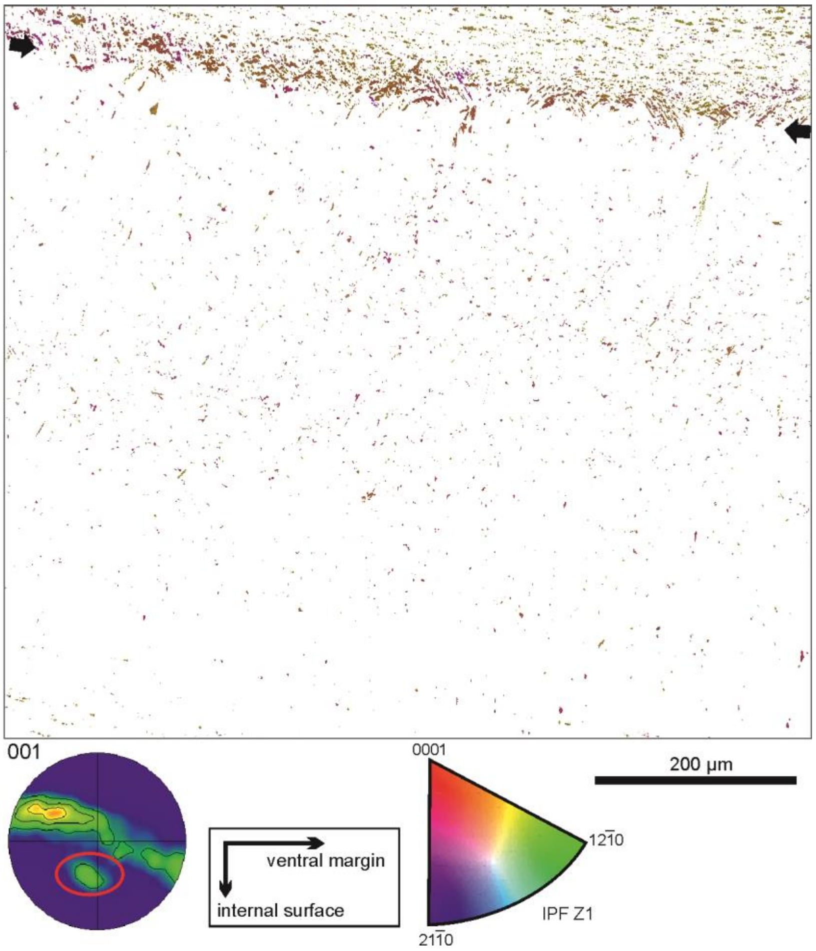

Supplementary Fig. S2. Persistence of elements of the foliated layer into the chalk. Same map as in Fig. 5 (foliated microstructure to the top), in which only the elements with a crystallography similar to that of the foliated layer (i.e. 001 poles within the encircled area) are shown. Despite their sparse, spotty distribution foliated-like elements go through the whole thickness of the chalk. Arrows indicate the boundary foliated layer-chalk. IPF, inverse pole figure (color key).

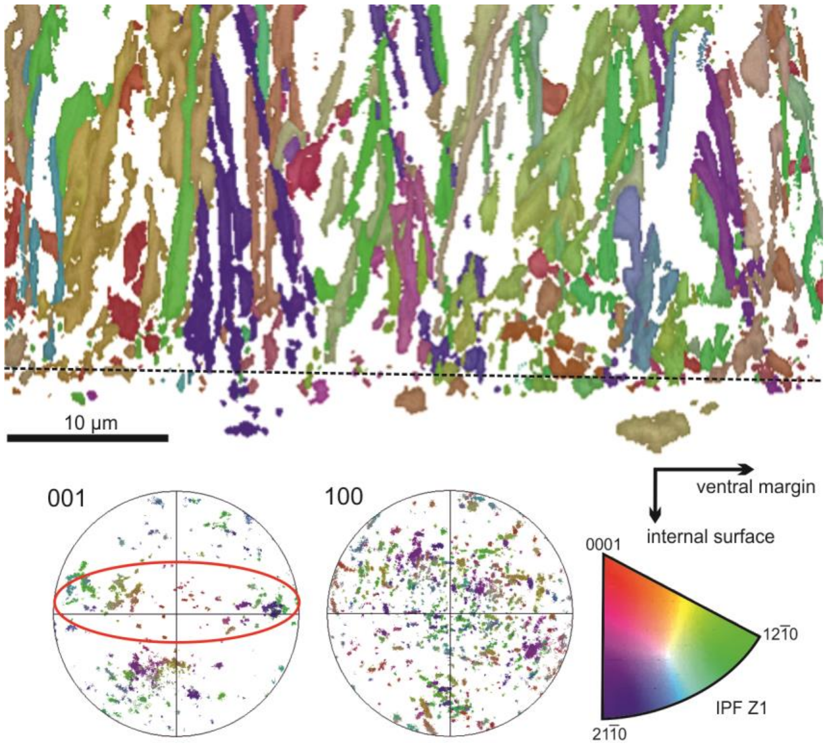

Supplementary Fig. S3. Sudden disappearance of chalk elements within the foliated layers. Same orientation map as in Fig. 4d in which only the elements with a chalk-like crystallography (equatorially distributed 001 poles, encircled) have been retained. The chalk elements do not appreciably intrude the foliated layer. The boundary between the chalk and the foliated material is indicated with the broken line. IPF, inverse pole figure (color key).

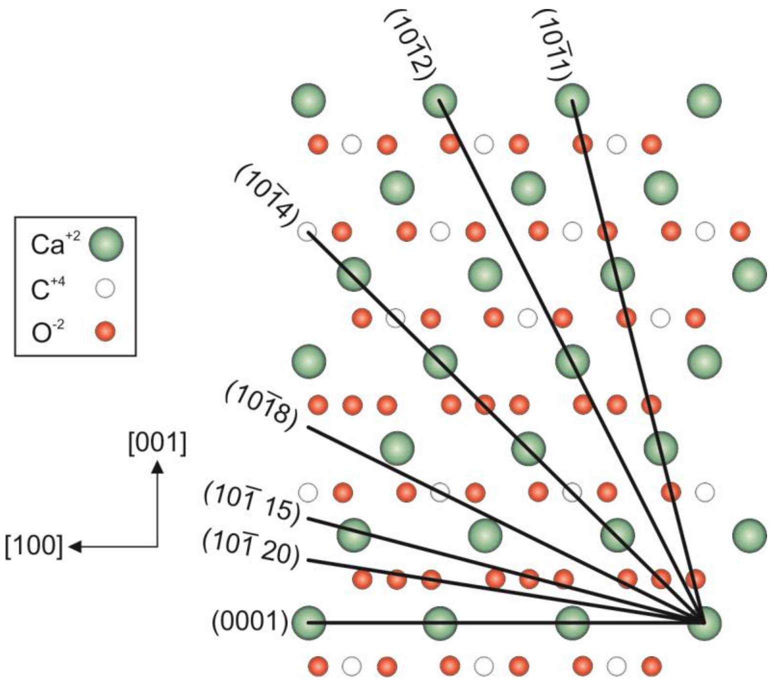

Supplementary Fig. S4. View of the calcite lattice along the *a*₂-axis, and the orientation of different rhombohedral faces (perpendicular to the viewing plane). Note the progressively lower number of atoms intercepted by the rhombohedral faces as the *l* Miller index increases from 4 [i.e. (10$\bar{1}$4)] to 20 [(10$\bar{1}$ 20)]. The rhombohedral face with the highest density (i.e. the most stable) is (10$\bar{1}$4).

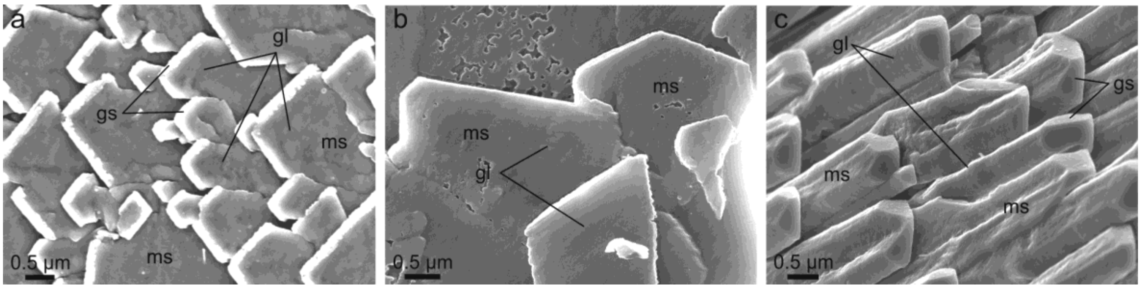

Supplementary Fig. S5. Contrast between the texture of the main and growth surfaces of laths in ostreids and pectinids. While the main surfaces (ms) are rough and bear growth lines (gl), the growth surfaces (i.e. {10$\bar{1}$4} faces) (gs) are smooth. (**a**) *Magallana angulata* (Cacela Velha, Southern Portugal). (**b**) *Anomia ephippium* (El Ejido, Southern Spain. (**c**) *Propeamussium dalli* (Maldive Islands).

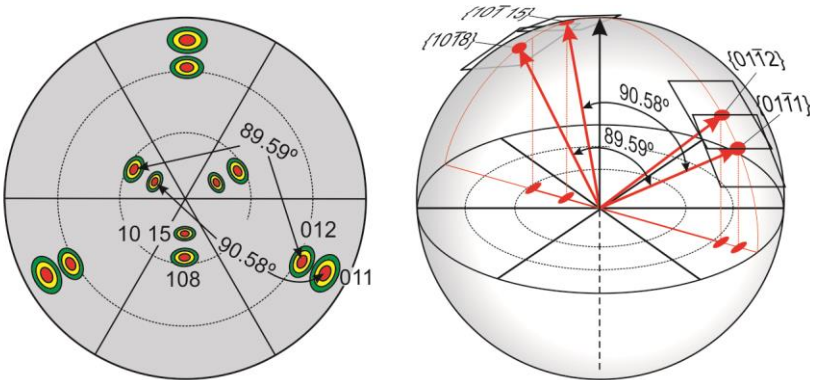

Supplementary Fig. S6. Quasi-perpendicularity between particular faces in calcite. The angular distances between 012 and 108 poles across the center of the pole figure is 89.59°, that is, the normal to the {01$\overline{1}$2} face is virtually contained within the {10$\overline{1}$8} face. Accordingly, 012 poles provide information about the orientation of {10$\overline{1}$8} faces. The same applies to 011 and 10 15 poles (and corresponding faces), separated by an angular distance of 90.58°.

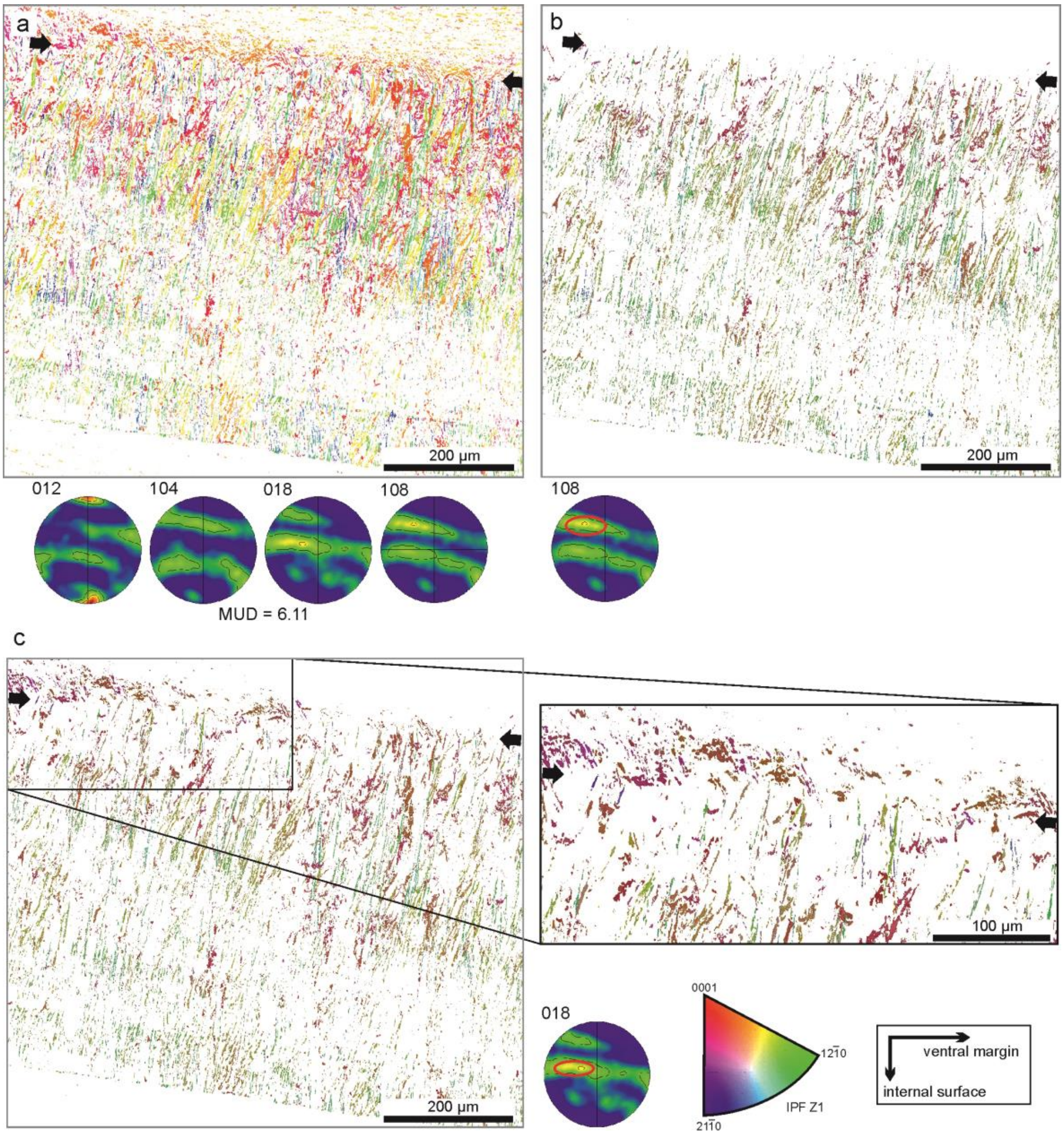

Supplementary Fig. S7. Hypothesized {01$\bar{1}$8} twinning relationship between elements of the foliated and chalk microstructures. (**a**) Orientation map (same as in Fig. 5) and pole figures corresponding to four out of the five twin planes defined in calcite (see text). (**b**) When the 108 maximum is cropped (red oval) we are left only with the elements (laths, blades, leaflets) having their 108 poles within the cropped area. In this case, all elements are within the chalk. The same happens when the 012 and 104 maxima are cropped. (**c**) Only when the 018 maximum is cropped we are left with elements of both the foliated layer and the chalk. This suggests that both materials share the corresponding (i.e. {01$\bar{1}$8}) calcite crystallographic plane. Boundaries between foliated and chalk layers indicated with arrows. IPF, inverse pole figure (color key).
